# Clinical Efficacy of an Eyedrop Containing Hyaluronic Acid and Ginkgo Biloba in the Management of Dry Eye Disease Induced by Cataract Surgery

**DOI:** 10.1089/jop.2021.0123

**Published:** 2022-05-03

**Authors:** Paolo Fogagnolo, Dario Romano, Valentino De Ruvo, Pierfilippo Sabella, Luca Rossetti

**Affiliations:** Eye Clinic, ASST Santi Paolo e Carlo, Università degli Studi, Milan, Italy.

**Keywords:** dry eye disease, cataract surgery, hyaluronic acid, ginkgo biloba

## Abstract

**Purpose:**

To evaluate the prevalence of dry eye disease (DED) after cataract surgery, and the impact of hyaluronic acid and ginkgo biloba eyedrops (HA-GB).

**Methods:**

Forty patients with no DED received Ocular Surface Disease Index (OSDI) questionnaire, assessment of conjunctival hyperemia and epithelial damage, fluorescein tear break-up time (TBUT) at baseline, day 1, week 1, and 4; adherence and tolerability were checked at weeks 1 and 4. At day 0 patients underwent cataract surgery and were randomized to standard postoperative care (control group) or standard postoperative care + HA-GB 3 times a day for 4 weeks (HA-GB group).

**Results:**

At baseline, TBUT was 9.6 ± 2.6 sec in controls and 9.0 ± 1.6 in HA-GB; thereafter it was higher in HA-GB group: 5.8 ± 2.3 versus 7.8 ± 3.2 (week 1, *P* = 0.03) and 6.4 ± 2.3 versus 8.5 ± 2.5 (week 4, *P* = 0.009). OSDI and conjunctival hyperemia were better in HA-GB group at week 4; respectively, 9.0 ± 5.7 versus 14.8 ± 7.3 (*P* = 0.004) and 5% versus 35% (*P* = 0.04). In the last 2 visits 50% of controls were symptomatic (OSDI of 13 or higher) compared with 16% on HA-GB group (*P* < 0.001). In addition, tolerability was higher in HA-GB group (week 1: 0.81 ± 0.20 versus 0.70 ± 0.24, *P* = 0.007; week 4: 0.93 ± 0.17 versus 0.80 ± 0.28, *P* = 0.001).

**Conclusion:**

Treatment with HA-GB is effective in reducing DED signs and symptoms in patients receiving cataract surgery, with high tolerability and safety profiles. clinicaltrials.gov (ID number NCT05002036).

## Introduction

Dry Eye Disease (DED) is a frequent clinical condition affecting from 14% to 33% of the population.^1^ Physiological tear film is a hydro-mucinic dispersion with a bilayer of lipids at the top. Classically, DED is divided in forms owing to low tear secretion (including Sjogren syndrome) and forms with increased tear evaporation.^2^ DED impairs the homeostasis of the eye surface, causing inflammation, abnormal osmolarity, and cell death in all tissues involved; this cycle tends to be self-perpetuating.^2^

Among the causes of DED, one of the most frequent is corneal nerve impairment, which may be owing to several causes: long-standing use of contact lenses, eye surgery (including refractive surgery), eye infections, and systemic diseases.^3^ Even cataract surgery, despite it being currently performed using very small incisions (1.8–2.4 mm), is associated with major changes to the nerve structures, which may sustain DED. In addition, suboptimal overlapping of the cut edges may also exacerbate the symptoms of DED.^3^ Finally, treatments used in the pre-, peri-, and postoperative periods (including antiseptic, antibiotic, and anti-inflammatory eyedrops) may damage conjunctival goblet cells, thus reducing tear film quality and quantity.^4^ As a matter of fact, modern cataract surgery induces transient DED with a prevalence of at least 10%.^5,6^

Current therapeutic options for DED treatment include lubrication with tear film substitutes and the management of local and systemic risk factors. Tear substitutes are of key importance in the treatment of DED, even if the resolution of other aspects of the pathology (including inflammation and eyelid pathologies) is also very important to attain successful treatment, especially in the most severe cases.^2^ Several options are available in clinical practice. Classically, hyaluronic acid (HA) has been considered as the most effective lubricating eyedrop for all types of DED, including postsurgical DED.

Other treatment options include lipid eyedrops or spray, lubricating gels or ointment, anti-inflammatory eyedrops (including steroids and cyclosporine), which are usually used to reduce both tear evaporation and the inflammatory component of DED, but are not a first line treatment for cataract surgery-induced DED.

In the recent years, many lubricating eyedrops have been enriched with several molecules (amino acids, vitamins, antioxidant) promoting ocular surface healing and reducing oxidative stress and inflammation that frequently play a key role on DED pathogenesis. In this study, we used a preservative-free formulation of 0.15% hyaluronic acid and 0.05% ginkgo biloba (HA-GB; Trium free eyedrops, Fidia srl, Italy). This medical device is used in the treatment of all DED and ocular surface epithelial damage, thanks to the protective effect of HA and to the antioxidant effect by GB.

There is a discrepancy between the high number of commercially available lubricating eyedrops and the poor number of studies exploring their clinical usefulness. Little evidence is currently available on the efficacy of lubricating eyedrops in reducing DED induced by cataract surgery. The aim of our study was to report the prevalence of cataract surgery-induced DED, and to investigate the clinical impact of HA-GB in managing it.

## Methods

This was a single-center, postmarketing, randomized, open-label, 4-week prospective study. It was conducted at ASST Santi Paolo e Carlo (San Paolo Hospital, Università degli Studi, Milan, Italy) after IRB approval (Comitato Etico Milano Area 1, No. 003018, 23/12/2020) from February to May 2021. The study followed the principles of the Declaration of Helsinki, the International Conference on Harmonisation guidelines on Good Clinical Practice (ICH E6), and ISO 14155; signed informed consent was obtained from all patients. The study was registered at clinicaltrials.gov (ID number NCT05002036). Patients and the public were not involved in any way in the research.

Forty consecutive patients needing cataract surgery were included in this study.

Inclusion criteria were as follows:

- Age of at least 18 years
- Cataract requiring surgery
- Tear break-up time (TBUT) of 7 s or more in both eyes
- Corneal staining of 1 or less using Oxford scale in one or both eyes
- Schirmer test >10 mm/5 min.

Exclusion criteria were as follows:

- Presence of corneal neuropathy (Cochet–Bonnet esthesiometry <50 mm) in one or both eyes
- Contact lens wear <30 days before surgery
- Autoimmune diseases
- Past or active ocular surface diseases (any corneal disease, cicatricial conjunctivitis, ocular surface burns, keratinization of the eyelid margin, Sjogren syndrome, corneal trauma)
- Pregnant and lactating women
- Ocular or general factors predisposing the patient to an increased risk for intraoperative complications, according to investigator's evaluation. These included pseudoesfoliatio capsulae, complete cataract, iridodonesis, previous eye surgery, previous eye trauma, history of complicated cataract surgery in the fellow eye, benign prostatic hyperplasia under treatment.

Patients were enrolled before surgery at baseline (−30 to −7 days from surgery); they underwent cataract surgery (day 0), and then were seen (according to normal clinical practice) at day 1 and at weeks 1 and 4.

At each visit, the following examinations were performed in both eyes in the following order:

- Ocular Surface Disease Index (OSDI) questionnaire
- Anterior segment ophthalmoscopy with grading of conjunctival hyperemia
- Fluorescein TBUT
- Grading of fluorescein corneal staining (epithelial damage)
- At week 1 and week 4, treatment adherence was checked and patients were asked to fill in a visual analogue scale for tolerability of study treatments.

Details of study procedures are given in Box 1. At baseline patients were checked for inclusion and exclusion criteria and the day of surgery they were randomized in an open way into 2 groups of postoperative treatments: group 1, 20 patients, standard care (dexamethasone + gentamicine eyedrops 4 times daily for 2 weeks, diclofenac eyedrops for the following 2 weeks); group 2, 20 patients, standard care + HA-GB 3 times daily. The characteristics of HA-GB are given in Table 1.

**Table 1.** Composition, Properties, and Physical Characteristics of Trium Free Eyedrops

Components	0.15% hyaluronic acid (properties: lubricating, promoting wound healing, anti-inflammatory)
	0.05% ginkgo biloba hydroglycolic extract (properties: promoting normal tear secretion, antioxidant, anti-inflammatory, promote axonal regrowth, relief postoperative pain)
	Isotonic-balanced electrolyte solution, buffered pH 7.2
Excipients	boric acid, sodium tetraborate decahydrate
Viscosity	6 to 14 cST
Osmolarity	270 to 230 mOsm/kg
pH	7.0 to 7.4
Average molecular weight	1,100,000 to 1,700,000 Da
Batch numbers	D15910 (6 bottles, expiry date: June 3, 2022) and D25420 (34 bottles, expiry date: June 5, 2022)

Cataract surgery was performed using a 2.4 temporal incision, with foldable intraocular lens in bag insertion.

The primary objective of the study was to test whether the postoperative treatment with HA-GB is effective in reducing the frequency of both signs (TBUT) and symptoms (OSDI) of DED induced by cataract surgery. The secondary objective was to evaluate the tolerability of study treatment.

The endpoints of the study were TBUT and OSDI (primary endpoints); number of patients with TBUT of 5 s or less, number of patients with corneal epithelial staining, tolerability of study treatments (secondary endpoints); and conjunctival hyperemia (safety endpoint).

Box 1.Diagnostic Procedures of the Study

Ocular Surface Disease Index

Ocular Surface Disease Index (OSDI) questionnaire (Copyright 1995, Allergan, Inc., Irvine, CA) was administered to the patient by a nurse who helped in filling in the answers without interfering on patient's judgment. OSDI score ranges from 0 to 100 (0–12, normal; 13–22, mild dry eye disease [DED]; 23–32, moderate DE; 33 or more, severe DED).^7^

Conjunctival hyperemia

Bulbar and tarsal conjunctiva was inspected at the slit lamp with 10 × magnification and white light; hyperemia was graded according to Efron et al.^8^ (0, normal; 1, trace; 2, mild; 3, moderate).

Tear break-up time

Tear break-up time (TBUT) was measured by determining the lapse between end of blinking and tear break-up. TBUT was performed after instillation of 2 μL of 2% preservative-free sodium fluorescein solution into the inferior conjunctival cul-de-sac of each eye. To thoroughly mix the fluorescein with the tear film, the patient was instructed to blink several times. To achieve maximum fluorescence, the examiner waited ∼30 s from instillation before evaluating TBUT. With the aid of a slit lamp at 10 × magnification using cobalt blue illumination, the examiner monitored the integrity of the tear film, noting the time it took to form lacunae (black spaces in the fluorescent tear film) from the time that the eye was opened after the last blink.

TBUT was measured twice during the first minute after the instillation of the fluorescein. If the 2 readings differ by more than 2 s, then a third reading was taken. The TBUT value was the average of the 2 or 3 measurements.^9^

Corneal fluorescein staining

Corneal fluorescein staining was assessed immediately after TBUT. Reading was performed between 1 and 4 min after fluorescein instillation, to ensure that the dye did not diffuse into stroma, blurring the discrete margin of any staining defects. The eye was examined at the slit lamp (16 × magnification) using a yellow barrier filter and cobalt blue illumination. Staining was graded using the Oxford scale.^9^

Study adherence

At weeks 1 and 4, adherence to treatment was checked by asking the patients if they used their medications correctly. Protocol treatment violation was defined in case of more than 2 consecutive missed doses or more than 3 missed doses per week.

Tolerability of study products

At weeks 1 and 4, patients were asked to define tolerability of study products using a visual analogue scale from 0 to 10, which was used to calculate this parameter as a ratio between 0 (absent tolerability) to 1 (maximum tolerability).

Being a strict classification of DED according to TBUT absent, we chose to include patients with TBUT of 7 s or more because these patients are generally asymptomatic, and this would have allowed us to quantify the percentage of patients showing DED symptoms after surgery. A subanalysis of TBUT of 5 s or less was carried out because this is a generally accepted cutoff to define moderate DED.

### Statistical analysis

The sample size estimate was based on a test for superiority for prospective studies (paired *t*-test), defining a 2-s change in break-up time as clinically relevant and a standard deviation of 2.3 s.^10–12^ Setting the error to 2.5% (one-tailed), 20 patients per group would be required, assuming a power of 90%.

An intent-to-treat approach was used to analyze signs and symptoms. In case of missing data, the last observation available was carried forward. Data were analyzed by means of *t*-test for paired data and Fisher exact test. Dataset was analyzed with R (R Foundation for Statistical Computing, Vienna, Austria)

## Results

All patients concluded the study with no missing data or treatment violations.

At baseline age, sex, preoperative best-corrected visual acuity (BCVA), and laterality were similar in the 2 groups: patients in HA-GB group were 73.1 ± 9.3 years of age; female/male ratio was 10/10, right/left ratio was 14/6, and BCVA was 0.43 ± 0.17; patients in control group were 73.4 ± 7.1 years of age (*P* = 0.75), female/male ratio was 7/13, right/left ratio was 9/11, and BCVA was 0.38 ± 0.21 (*P* = 0.44).

Mean data of efficacy in the 2 groups at each visit are given in Table 2 (TBUT) and Table 3 (OSDI). Both parameters had a statistically significant amelioration in HA-GB compared with control group both at weeks 1 and 4. In the last 2 visits, 50% of controls were symptomatic (OSDI of 13 or higher) compared with 16% in HA-GB group (*P* < 0.001).

**Table 2.** Tear Break-Up Time During the Study

	HA-GB group	Control group	*P*
Baseline (s)	9.0 ± 1.6	9.6 ± 2.6	0.38
Day 1 (s)	5.8 ± 2.4	6.8 ± 4.1	0.33
Week 1 (s)	7.8 ± 3.2	5.8 ± 2.3	**0.03**
Week 4 (s)	8.5 ± 2.5	6.4 ± 2.3	**0.009**

Values in bold indicate *P* < 0.05.

HA-GB, hyaluronic acid and ginkgo biloba.

**Table 3.** Ocular Surface Disease Index Scores Over the Study

	HA-GB group	Control group	*P*
Baseline	10.3 ± 8.0	8.7 ± 4.4	0.52
Day 1	13.3 ± 8.3	12.9 ± 7.3	0.97
Week 1	9.0 ± 5.7	14.8 ± 7.3	**0.004**
Week 4	6.4 ± 3.5	14.6 ± 7.3	**0.0001**

Values in bold indicate *P* < 0.05.

Percentage of subjects showing BUT = 5 s or less at each visit (Table 4) ranged from 5% to 10% in HA-GB group and 15%–25% in control group, although the difference was not statistically significant.

**Table 4.** Percentage of Patients with Tear Break-Up Time of 5 s or Less

	HA-GB group, %	Control group, %	*P*
Baseline	0	0	1.0
Day 1	80	70	0.71
Week 1	10	25	0.40
Week 4	5	15	0.23

The trend of conjunctival hyperemia is given in Table 5. In control group, the percentage was constantly higher than HA-GB (respectively, 35%–60% vs. 5%–40%) and at week 4 HA-GB group had significantly less hyperemia than controls (*P* = 0.04).

**Table 5.** Percentage of Patients with Conjunctival Hyperemia

	HA-GB group, %	Control group, %	P
Baseline	25	10	0.40
Day 1	70	50	0.33
Week 1	40	60	0.34
Week 4	5	35	**0.04**

Values in bold indicate *P* < 0.05.

Also corneal epithelial damage (Table 6) was lower in patients treated with HA-GB (10%–35% vs. 35%–60% in control group), although the differences were not significant.

**Table 6.** Percentage of Patients with Epithelial Damage

	HA-GB group, %	Control group, %	*P*
Baseline	0	0	1.0
Day 1	60	50	0.75
Week 1	35	60	0.20
Month 1	10	35	0.21

During the course of the study, no statistically significant modifications occurred in any parameter in the fellow eye. Adherence to treatment was very good in both groups (self-reported adherence was 0.95% in both groups). Tolerability in patients of HA-GB group was significantly higher than control group both at weeks 1 and 4 (week 1: 0.81 ± 0.20 vs. 0.70 ± 0.24, *P* = 0.007; week 4: 0.93 ± 0.17 vs. 0.80 ± 0.28, *P* = 0.001).

## Discussion

This study evaluated the impact of uneventful cataract surgery on the prevalence of DED-related symptoms on a population without preoperative DED. We confirmed that even a minimally invasive approach with small incisions actually induces relevant changes to the ocular surface at least in the first postoperative month. In control group, mean TBUT decreased by 30%–40% owing to surgery; at the end of follow-up, a mean reduction of 3.2 s compared with baseline was shown. In control group, 25% and 15% of patients had TBUT of 5 s or less after surgery; epithelial damage and conjunctival hyperemia were present after surgery in 35%–60% of patients, despite the use of topical anti-inflammatory eyedrops. Overall, controls were symptomatic in 50% of cases within 1 month after surgery.

The postoperative use of HA-GB given 3 times daily was effective in improving both signs and symptoms of DED. Mean TBUT was significantly higher than control both at week 1 and week 4, with a mean increase of ∼2 s between day 1 and week 1; at the end of follow-up, TBUT in these patients was just 0.5 s lower than baseline and only 2 patients had TBUT of 5 s or less. Also, conjunctival hyperemia and epithelial damage progressively improved: at week 4 these parameters were positive in just one patient. At the end of the study conjunctival hyperemia was significantly lower in HA-GB than in control group.

Symptoms were assessed with OSDI; in control group, cataract surgery induced only a mild worsening of symptoms, but 50% of patients were symptomatic at weeks 1 and 4 (OSDI higher than 13). On the contrary, patients using HA-GB had postoperative scores even lower than before surgery, and only 16% at abnormal OSDI scores.

The use of HA-GB significantly increased the tolerability of postoperative medications compared with control group.

The interpretation of the results of this study would vary if different cutoffs were used, particularly for TBUT. In general, a TBUT cutoff of 10 s or less is accepted to define the presence of DED,^9^ although this definition may include a large number of asymptomatic patients or even a large prevalence of non-DED subjects.^13^ As a matter of fact, we included patients with TBUT of 7 s or more at baseline, and they had no DED symptoms according to OSDI score. Lower cutoffs are therefore better descriptors of symptomatic DED, and in particular the cutoff of 5 s or less used to describe postoperative data are considered an indicator of short TBUT DED^14^ and it is valid regardless of the amount of fluorescein used^15^; in many settings, TBUT of 5 s or less is also an accepted cutoff to define moderate DED.

We studied a population with no DED at inclusion to evaluate how many subjects develop postsurgical DED. Different results would have been obtained in patients with preexisting DED or risk factors for it. In our population, HA-GB was beneficial in our group of patients and we may raise the hypothesis that even better results would have been obtained in patients already symptomatic at baseline.

Despite DED-related symptoms being relevant for patients undergoing cataract surgery, this problem is relatively poorly explored. It is possible that similar results in our study could be obtained also with other lubricating eyedrops. HA alone is in fact effective in reducing epithelial damage and improving TBUT after cataract surgery,^16^ and we previously showed a beneficial effect after cataract surgery also using a combination of hypromellose and coenzyme Q10^17^ and a combination of liposomes, omega 3, vitamin D and vitamin A palmitate^18^; also diquafosol^19^; and lifitegrast^20^; showed beneficial effects.

A recent meta-analysis quantified the amelioration of TBUT after a 1-month treatment with HA to ∼2.0 s (95% confidence interval, 1.63–2.49 s).^16^ In our study, the mean change occurring between day 1 and week 4 in HA-GB was higher (2.7 s). The higher success of our study may be explained by the inclusion of patients with less severe ocular surface damage compared with other studies or with an extra benefit owing to the beneficial properties of GB in DED management (thanks to the antioxidant and anti-inflammatory properties, GB promotes normal tear secretion; moreover it also promotes axonal regrowth and reduces postoperative pain).^21–23^

Only a head-to-head randomized study comparing HA alone with HA-GB may quantify the advantage of gingko biloba in DED, although the superiority of HA-GB to HA alone was already shown by Russo et al. on patients with allergic conjunctivitis.^24^

This study may be limited by the following factors. Study population was small, although sample was coherent with study assumptions and size calculation. Open randomization could have affected results and blindness of the evaluators would have been desirable. In addition, the use of a third group using HA alone would have been useful to better clarify the impact of GB on DED. Yet it should be considered that HA with similar characteristics of the study product is not commercially available; the use of HA with different concentrations and molecular weight would have added further noise to data interpretation.

TBUT assessment is prone to large test-retest variability and inter-evaluator agreement may be low. In patients receiving cataract surgery, the high prevalence of dry spots may also affect a correct assessment of TBUT. In this study, we used trained evaluators and standard operating procedures; the coherence on the prevalence of signs and symptoms also seems to corroborate the lack of bias on data collection. Yet, we are aware of the possible limits of the assessment of TBUT and, more in general, of nonautomated measures for DED. Finally, further investigation is warranted to evaluate the effects of HA-GB compared with different lubricating eyedrops in surgical-induced DED.

In conclusion, our study showed that cataract surgery is associated with relevant OS changes occurring at least for the first postoperative month, and that the treatment with HA-GB is highly effective in reducing DED signs and symptoms, with a high tolerability and safety.

## Data Sharing Statement

Data available upon request to corresponding author.
